# Coping Strategies To Enhance Mental Health and Wellbeing of Ethnic Minority Populations in the United Kingdom: A Scoping Review

**DOI:** 10.1007/s10903-025-01761-3

**Published:** 2025-08-25

**Authors:** Rajeeb Kumar Sah, Devendra Raj Singh, Bibha Simkhada, Lalita kumari Sah, Jenny Retzler, Tracey Smith, Wajid Khan, Michael Doyle

**Affiliations:** 1 University of Huddersfield, Huddersfield, United Kingdom; 2 University of Bradford, Bradford, United Kingdom; 3 University of Leeds, Leeds, United Kingdom; 4 South West Yorkshire Partnership NHS Foundation Trust, Wakefield, United Kingdom; 5 University of Manchester, Manchester, United Kingdom

**Keywords:** Coping strategies, Mental health, Wellbeing, Ethnic minority

## Abstract

Mental health issues among ethnic minority populations in the UK are a significant concern. Synthesised evidence related to coping strategies to improve mental health among these groups is lacking. This scoping review aimed to identify and consolidate literature on coping strategies used by ethnic minority groups in the UK to overcome their mental health problems. This review reflects a strengths-based approach that emphasises how ethnic minority populations deploy coping strategies in response to mental health challenges, rather than merely focusing on barriers. This scoping review was guided by the methodological framework provided by Arksey and O’Malley. Literature was searched in MEDLINE, CINAHL, PsycINFO, and Science Direct databases. The review result was reported following the PRISMA extension for Scoping Reviews (PRISMA-ScR) Checklist. A total of 2888 records were identified from the database search after removing the duplicates, and 17 records were included in the review. Different coping strategies and their barriers and challenges were identified and presented under three primary themes: (i) Self-help and immediate personal and social support networks, (ii) Community-based and professional mental health services, and (iii) Challenges and barriers. A broader understanding of the community strengths and resources of ethnic minorities and their adequate integration with mental health services can strengthen the existing efforts in improving the mental health of ethnic minority populations in the UK.

## Background

The mental health and well-being of ethnic minority groups in the United Kingdom (UK) are concerning despite several efforts made by the government and other sectors [1]. Mental health problems among ethnic minorities are relatively higher compared to the white British population in the UK [2–4]. In the UK, the term ethnic minority refers to the population of Black, Asian, Mixed, and other non-White British minority groups [5], which comprises 18% of the total UK population [5]. These populations experience challenges at different levels that adversely impact their mental health and well-being. The most common challenges among these groups, which are widely documented, are experiences of discrimination, racism, acculturative stress, socioeconomic inequalities and sociocultural and language barriers in accessing mental healthcare services [1–3, 6, 7]. Results from a survey in England showed the ethnic minority population experienced 17.9 to 22.5% of common mental disorders compared to [2–4, 6, 7] general population [8]. Further, several studies show that ethnic minority populations may also have poor access to mental health services, and often, they have more negative experiences about coping with their mental health issues [2–4, 6, 8]. In most cases, their mental health problems remain undiagnosed or are identified at the later stages of the mental illness through crisis or deterrent pathways [4]. Multiple factors are reported as barriers among ethnic minority populations for accessing mental health services, such as poor knowledge and acceptability of mental health issues, reluctance to discuss and seek help related to mental health problems, cultural differences, language barriers, discrimination towards mental health needs of minority groups, power imbalance between service providers and service users, and difficulty in navigating through the health system [2–4, 7, 9]. Thus, these groups of populations often require support and an environment that facilitates a strength-based coping approach, aligning with their individual and community strengths, encouraging them to take an active role in promoting their mental health and well-being [10].

Early identification and treatment of emerging mental health problems is crucial to reduce their deterioration and adverse impact on the well-being of the population [10]. Thus, individuals need to understand how to recognise the risks associated with their mental health, identify and use preventative approaches to minimise those risks and understand how to seek additional help [11, 12]. These skills are often referred to as “coping strategies”, a term that encompasses the actions, behaviours, emotions, and thoughts that a person uses to deal with stressful circumstances [13]. Such thoughts and behavioural skills, which individuals use to manage their stressful situations, are based on the demand for actions in response to internal or external factors [13, 14].

The concept of coping mechanisms is complex, and several authors have illustrated various strategies for coping [13]. Folkman and Moskowitz [14] described four categories of coping strategies [14]. The first coping approach is problem-focused, where individuals address the causes of distress through active planning and suppression of risks by competing actions. Second, is the emotion-focused coping approach, where a person reframes their situation in such a way that they adopt various actions such as religious, spiritual and humour to create a favourable environment for themselves. The third approach is meaning-focused, where individuals act based on their cognitive skills to deal with the situation. Fourth, coping through social support or help-seeking, where individuals utilise social and community support mechanisms to overcome their adverse circumstances. Building on this, the authors discussed various new developments in coping research, including social aspects of coping, religious coping, and emotional approach to coping. To simplify the concepts of coping strategies in this review, we synthesise our review findings to align with these three approaches to coping.

In addition to the mental health inequalities mentioned, there are also disparities within ethnic minority groups in relation to the coping strategies used by them at the individual level and towards accessing community mental health services [3, 4, 6, 15–17]. However, it is not explicit how these variations exist within and across various ethnic groups, nor how effective they may be for dealing with mental health problems. Literature shows that individual coping strategies are shaped by an interplay of individual, sociocultural and wider environmental factors [4]. However, the synthesised evidence from the existing literature relating to coping strategies, particularly those used among ethnic minorities within the UK context, is lacking. This evidence synthesis of coping strategies used by individuals from ethnic minority backgrounds provides a comprehensive understanding of the diverse knowledge, skills, and broader environmental circumstances in which they practice certain types of coping strategies in dealing with their mental health issues. Thus, this scoping review aims to identify and synthesise literature on coping strategies used by minority ethnic groups in the UK to overcome their mental health problems.

## Methods

The methods for this scoping review were guided by the five-step methodological framework provided by Arksey and O’Malley’s approach to scoping review [18]. We employed an iterative team approach where regular sharing and discussion about the review progress took place in team meetings among reviewers to ensure consensus and clarity on the next steps in the review process. In addition, to enhance the rigour and robustness of the review results, we also conducted a validation workshop among multi-stakeholders as a consultation exercise. Although an optional stage of the Arksey and O’Malley scoping review framework, we believe that adding a stakeholder consultation exercise as the sixth step to inform and validate findings from the main scoping review greatly enhanced our work and significantly enriched the process and outcomes of the review. We emphasise making stakeholder consultation a mandatory component of the methodology, which provides opportunities for sharing the initial findings from the review and incorporating stakeholders’ feedback to fill the gaps in unexplored aspects of the review [18, 19]. The review results were reported following the PRISMA extension for Scoping Reviews (PRISMA-ScR) Checklist [20]. The six steps of the review process are described below:

### Step 1: Identifying the Research Question

This review was guided by the research question: What types of coping strategies are used by ethnic minority populations to enhance their mental health and well-being? Coping strategies related to mental health are broadly referred to as the thoughts and behaviours mobilised to manage internal and external stressful situations [14]. Coping strategies could include a wide range of actions, such as problem-focused, emotion-focused, meaning-focused, and social support or help-seeking [14]. However, there is debate about whether one strategy is more beneficial than another in coping with mental health issues. Considering the purpose of the review and the potential for adopting a comprehensive range of coping actions in mental health, we included all possible concepts of coping strategies.

### Step 2: Literature Search

To identify and retrieve relevant studies, a comprehensive search was conducted across the four databases: MEDLINE, CINAHL, PsycINFO, and ScienceDirect. The key concepts and search terms were combined and expanded using Boolean Operators (AND, OR) to search the literature (Table 1).

**Table 1:** Search terms and concepts

Key Concepts	Search Terms
Coping strategies	Coping OR “formal support” OR “informal support” OR help seek* OR seek* help OR seek* treatment OR “problem-solving” OR “support seeking” avoidance OR distraction OR “positive cognitive restructuring”
Ethnic minority	Ethnic Groups OR Minority Groups OR African Continental Ancestry Group OR Asian Continental Ancestry Group OR minority ethnic OR Black and minority ethnic OR Black, Asian and minority ethnic OR ethnic minoriti$ OR people of colo?r OR Black, Indigenous and people of colo?r OR Black African$ OR African Caribbean OR African American$ OR Pakistani$ OR Indian$ OR Bangladeshi$ OR South Asian$ OR East Asian$ OR South East Asian$ OR BME OR BAME
Mental health	Mental Health OR Mental disorder* OR Mental OR mental illness* OR “mental well-being” OR “Mental distress” OR “Mental Health and well-being” OR psychiat* OR depress* OR anxiety

### Step 3: Screening and Selection of Studies

**Fig. 1:**
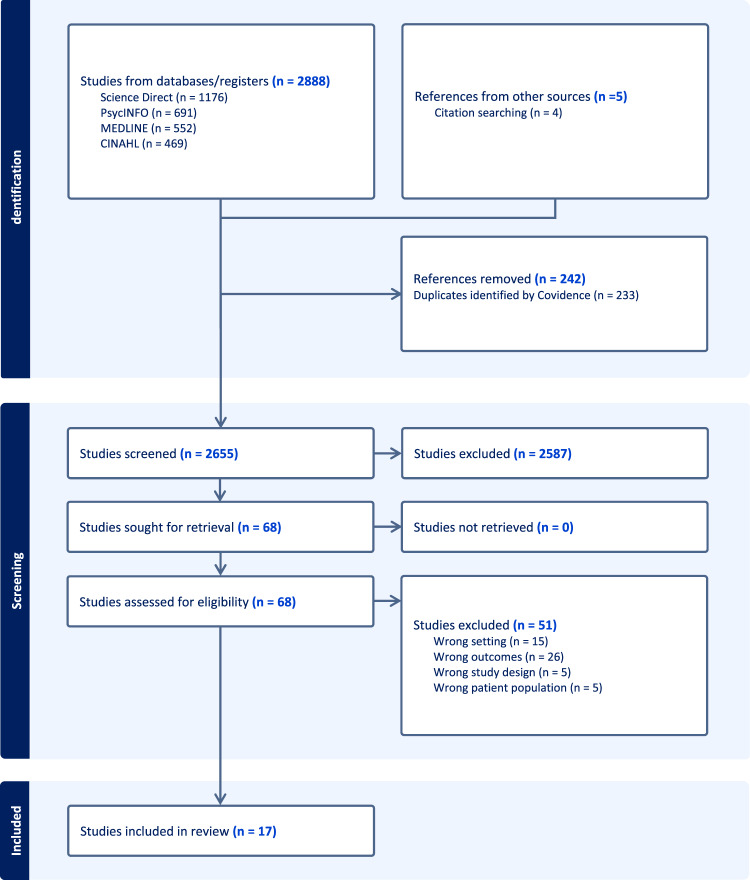
PRISMA chart

The review included all empirical research articles written in the English language, research conducted in the United Kingdom and published before December 2023. Considering the limited evidence in this area of research among minority populations in the United Kingdom, we decided to include all relevant published papers prior to December 2023 in this review. Research papers reporting coping strategies related to mental health and well-being among the ethnic minority populations of the United Kingdom were included. We considered all types of study designs. However, we excluded those studies reporting coping strategies related to the mental well-being of mothers during the perinatal period, the geriatric population, those in health or other specific professions, those with specific health conditions, and those with severe forms of mental disorders. The identified records were transferred to EndNote, and after removing the duplicates, all indexed files were moved to Covidence Software for title and abstract screening. Two co-authors independently performed the title and abstract screening. Following a full-text review of selected papers, those meeting the inclusion criteria were included in the review. The record selection process has been presented in the PRISMA chart (Fig. 1).

### Steps 4 & 5: Data Charting and Reporting the Results

The data from the included papers were extracted into the predefined Microsoft Excel sheet with headings for: author/date, study site, study design, sample population, sample size, coping strategies, challenges and barriers related to coping strategies. The results were consolidated using a narrative synthesis approach and summarised under overarching themes.

### Step 6: Stakeholder Consultation

A consultation workshop was conducted among multi-stakeholders via Microsoft Teams. The summary of the review results was shared with participating stakeholders two weeks before the consultation workshop. A total of twelve stakeholders participated, including a community psychologist, a clinical psychologist, representatives from charities supporting community mental health, and academics and researchers in mental health, which ensured the diversity and inclusiveness of the stakeholders. The workshop shared the initial results from the scoping review for discussion and validation, and to incorporate stakeholder feedback on any gaps in the evidence relating to the review topic, thereby minimising discrepancies in the overall synthesised results.

## Results

A total of 2888 unique records were identified from the database search after removing the duplicates. Subsequent screening based on titles and abstracts identified 68 records for full-text review. Ultimately, 17 records met the defined inclusion criteria and were consequently included in the review.

## Characteristics of the Included Studies

Among the 17 studies included in this review, qualitative designs were employed in 13 papers [7, 15–17, 21–29], quantitative designs in two papers [30, 31] and mixed methods designs in two papers [32, 33], collectively investigating coping strategies within ethnic minority populations in the United Kingdom. Furthermore, ten papers presented findings representing mixed ethnic minority groups [7, 21, 25–30, 32, 33], with four papers focusing on African, Caribbean, and other ethnic minorities [16, 17, 22, 23], two papers on Asian British or Asian backgrounds [24, 31], and one paper exclusively reporting outcomes from Muslim religious groups [15]. Notably, 14 investigations recruited adults from ethnic minority communities [7, 15–17, 21, 22, 24–26, 29–33], while three studies were specifically conducted among children and adolescents within these populations [23, 27, 28]. The detailed characteristics of the studies included in the review are presented in Table 2.

**Table 2:** Characteristics of included studies

Author(s) (Date)	Study design	Study sites	Sample size (n)	Sample population	Coping strategies
Bhui et al. [21]	Qualitative	Natiide (UK)onw	n=116	Bangladeshi, Caribbean, Indian,Irish, Pakistani, and White British people	· Religious practices and rituals stoicism, a positive outlook, survival thinking, hopefulness, drawing upon lived experience, normalizing, rationalization, avoidance, distraction/escapism, crying, responsibility for others and relaxation
Bortel et al. [29]	Qualitative	Nationwide (UK)	Women=5, Men=14 Non-binary=1	Ethnic minorities	· Physical exercise, walking, cycling, home workout, keeping in touch with friends and family. · Having support systems from different networks of life, friends, family, work, neighbours, religious community, spiritual practices, eating more, and alcohol consumptions
Dare et al. [22]	Qualitative	UK (not specific)	n=6	African, Caribbean, and similar ethnicity (ACE)	· Seeking help from a professional · Avoidance · Religious · Consulting GP and other clinicians
Dewa et al. [32]	mixed methods	Nationwide (UK)	n=796, n=18	Black, Asian, and Minority Ethnic	· Emotion-focused coping such as controlled breathing, mindfulness, and meditation · distracted themselves in various ways to help keep themselves busy and take their minds off issues · Played music, podcasts, or watched Netflix positive reframing to cope and being spontaneous, optimistic, and joking with friends or family · Consulting GPs and other clinicians
Gunasinghe, et al. [15]	Qualitative	London, England	n=6	Muslim	· Tolerating distress and isolation · Considered endurance as a strength · Acceptance · Seeking help form family and friends · Practice cultural and religious approaches · Help-seeking for psychological distress through external agencies
Halliday et al. [33]	mixed methods	Northwest England	n=15	White British, Pakistani, Black African and White, not stated.	· Mindfulness and self‐help techniques · Complete normal routine · Finding meaning and sense making · Volunteering · Jogging · Meditation
Lai et al. [30]	Quantitative	UK and USA	n=124	Asian and non-Asian	· Listening to music, eating, or cooking, video or mobile gaming, seeking support from family and friends, browsing the web, positive thinking, exercise, religious support, and meditation
Lenoir and Wang [23]	Qualitative	West London, England	n=10	Black or black African (n=4), Black or black Caribbean (n=1), any other Black background (n=1), white and Black African (n=2) and white and Black Caribbean (n=1)	· Over-thinking · Release of aggression · Reading · Self-evaluation · Maintaining a healthy routine
Martinez et al. [24]	Qualitative	London, England	n=124	Filipino migrants	· Cognitive reframing · Religious coping such as praying · Distracting or keeping oneself busy or doing something else · Seeking informal help · help-seeking from friends, family, partners, and community organisations/charity organisations. · Help from social networks · seeking professional assistance for their mental health service providers
Meechan, John and Hanna [16]	Qualitative	South London, England	n=10	Black Caribbean	· Seek help from professional mental health service providers · Religious · Friends
Memon et al. [7]	Qualitative	Southeast England	n=26	Black, Asian, and Minority Ethnic	· Family and friends · Seek support from social networks
Mantovani et al. [17]	Qualitative	South London, England	n=26	Faith‐based African	· Religious based · Spiritual practices · seek help from mental health professionals
Kumari [25]	Qualitative	UK (not specific)		Mixed ethnic population	· Religious and cultural approach · Seek help from mental health professionals
Ogueji et al. [26]	Qualitative	UK (not specific)	n=52	Mixed ethnic population	· Socializing with loved ones through video calls, engaging in exercise, being occupied with jobs, being occupied with studies, avoiding negative news, consumption of alcohol, healthy eating, engaging in meditation activities, gaming activities, hope, and self-care and self-appreciation
Stapley et al. [28]	Qualitative	England (Blackpool, Cornwall, Hull, Kent, Newham borough of London, and Wolverhampton	n=82	White British, White Irish, and Black, Asian, and Minority Ethnic	· Feeling sad or angry, such as by being in their bedroom, and walking away or removing themselves from arguments with parents, peers, and siblings · Accepting the problem, difficult situation, or feeling engaging in various creative activities, such as · Reading books, drawing, and colouring, acting or singing · Physical activities including going for a bike ride or a walk, boxing, playing football, or dancing · Digital or media entertainment, being physically active, Positive thinking or optimism, Perseverance,
Stapley et al. [27]	Qualitative	England	n=31	White British, and Black, Asian, and Minority Ethnic (adolescents)	· Engaging in activities, using technology, disengaging from difficulties, · Positive thinking, · Accepting difficulties · Self-defence · Support from both parents, support from one parent, support from other family · members. · Support from friends, support from school staff, Other professional support (support received from HeadStart or other professionals (e.g., child and adolescent mental health services; CAMHS)
Soorkia, Snelgar and Swami [31]	Quantitative	UK (not specific)	n=148	Indian, Pakistani, Bangladeshi, or other South Asian descent	· Religious and cultural approach · Support from both parents, support from one parent, support from other family · members · Seek help from mental health professionals

## Themes

Studies included in the reviews found that ethnic minority populations employed different coping strategies to enhance their mental health and well-being. These coping strategies could be broadly categorised as either personal-level or community-related. The barriers and challenges in adopting such strategies were apparent in both categories and are considered separately. Thus, the review results are presented under three overarching themes: (i) Self-help and immediate personal and social support networks, (ii) Community-based and professional mental health services, and (iii) Challenges and barriers.

### i) Self-help and Immediate Personal and Social Support Networks

Ten studies included in the review reported that participants’ close circles, such as parents, family members, friends, and members from their ethnic communities or organisations, were considered trustworthy individuals with whom they shared their mental health issues or emotional crises [15–17, 21, 23, 24, 27–33]. Further, three studies reported that adolescents often viewed their parents as consistently accessible and trustworthy figures to whom they could turn for advice and support regarding stressful life events at home and school [23, 27, 28]. Of note, a study presented that the coping strategy utilised by the head of the family, particularly fathers, is often the one adopted by others in the family [16]. Additionally, the roles of friends and siblings in the lives of adolescents were highlighted in recognising signs of distress and aiding them in seeking help from adults, such as their parents [28].

Similarly, adults were also found to seek help from their social networks, such as friends, family, partners, and third-sector charitable organisations, during emotional crises [17, 21, 23, 24, 29]. Participants in two studies felt at ease and comfortable disclosing their mental health problems to friends, as they often received supportive responses and felt encouraged to seek help, including services from local community organisations such as religious centres [24, 27]. Likewise, seven studies in the review reported that adopting or engaging in religious activities was one of the commonly practiced coping mechanism related to mental health [15, 21, 22, 24, 30, 32, 33]. Common religious coping strategies reported were practices like praying for guidance and strength, attending religious sites, listening to religious radio, using amulets, talking to God, and participating in religious rituals [15, 17, 21, 24, 29, 33]. Five out of these seven studies in the review illustrated religious or faith-based coping strategies, where participants were found to have relied on religion as a way of coping to assist in solving or mitigating their negative mental emotions in various life circumstances [15, 17, 24, 29, 33]. Participants in some studies expressed beliefs that religious actions help to manage precarious emotions caused by irreligious actions [21, 24, 29]. Similarly, some studies in the review reported variations in the religious coping strategies adopted among different ethnic and religious groups within the minority population [21, 24]. Two studies mentioned prioritising spiritual approaches over recommending seeking help from mental health professionals [17, 21].

In addition, eight studies reported adopting different types of creative activities as coping strategies to keep themselves distracted and busy doing activities that took their mind off the stressful situation [24, 26–30, 32, 33]. Six studies reported that engaging in different activities like drawing, playing video games or football, intentionally forgetting about the problems, choosing to mentally set aside issues, or deliberately ignoring the existence of problems and individuals causing distress served as a distraction to themselves, allowing individuals to shift their focus away from problems to relax and experience enjoyment [26–30, 32]. Studies reported that such activities helped participants redirect their thoughts and alleviate worries. Other studies highlighted the adoption of particular lifestyle choices as coping mechanisms, particularly making changes during periods of COVID-19 lockdown with initiatives such as eating well and regular exercise, quality sleep, outdoor time, controlled breathing, mindfulness, and meditation [26, 29, 30, 32]. Three studies also reported alcohol consumption as a mental coping strategy [26, 29, 32]. Likewise, using technologies to engage in video calls, listening or playing music and podcasts, and watching Netflix were commonly practised [27–30, 32]. A study among diarists presented that interaction with the environment, especially in good weather, contributed positively to the well-being of participants through walks and time in green spaces [33]. Elsewhere, participants reported that seeking entertainment and social connection, such as joining a migrant organisation and staying busy supporting others by volunteering in community organisations, helped distract them from their mental distress [24].

### ii) Community-based and Professional Mental Health Services

Another common coping approach was to seek formal and professional support by accessing mental health services present within the local community and clinical settings, such as consultation with General Practitioner (GP) and mental health professionals, psychiatrists, psychologists, guidance counsellors, and social workers [7, 16, 22, 24, 27–30, 32, 33]. Most studies included in this review highlighted that adult talked about accessing professional mental health services for treatment, with no/limited discussions about the skills-based approaches to manage or cope with their mental health. Two studies reported adolescents accessing Child and Adolescent Mental Health Services (CAMHS) and the HeadStart programme in the form of peer mentoring, one-to-one support, group psychoeducational programmes, and co-production activities, primarily happening at schools [27, 28]. It was discussed that these activities provided adolescents with the opportunity to solve their stressful issues related to schoolwork, exams, conflicts with friends, bullying, family problems, and managing various emotions like sadness, anxiety, and anger [27, 28]. Using stress balls and breathing techniques, positive thinking guidance, advice on problem-solving, and addressing bullying issues were other commonly practised activities. Adolescents considered the HeadStart settings as an “escape” or a safe space, as they can comfortably talk with HeadStart staff about their problems, either in addition to or instead of family, friends, and teachers. They felt peer mentors, being closer in age, were more approachable than adults and offered a unique perspective and advice different from that of their normal peers. Additionally, confidential emotional support was offered during one-to-one or group sessions. These papers also highlighted the availability of Child and Adolescent Mental Health Services (CAMHS) that were accessible to young people, providing services for assessing and treating their emotional, behavioural, and mental health difficulties [27, 28]. Few participants in one of these studies reported that they had been receiving services from both HeadStart programme and CAMHS. Both these services were accessed through referrals from charity organisations, social networks, friends, and community-based organisations [24].

### iii) Challenges and Barriers

Thirteen studies in the review discussed the different challenges and barriers ethnic minorities face when adopting coping strategies for mental health [7, 15–17, 21–24, 27–29, 31–33]. The evidence suggests significant concerns about access to and uptake of effective coping strategies. Barriers among those from minority ethnic backgrounds to seeking support from immediate personal and social networks and professional mental healthcare services included a lack of shared values and understanding of cultural competency or backgrounds [7, 15–17, 21, 24, 32]. The challenges and barriers experienced and perceived by ethnic minorities in coping with their mental health issues identified in the review are categorised under three levels: personal, societal, and institutional.

Several studies reported that professional support for mental health was considered as a last resort for individuals at a personal level; participants only sought help when difficulties reached a critical point and external help was necessary [16, 24, 33]. Likewise, perceived self-reliance and specific personality traits were also identified as significant barriers to seeking mental health support among the immigrant population [16]. However, the perception and experience of overcoming the most challenging problems gave them the confidence to be self-reliant and deal with certain stressful situations [16]. Hesitation in seeking help was reported to have potentially harmful consequences for the mental health of those relying solely on faith-based organisations for support [15, 17]. In addition, adolescents cited various other reasons for not seeking mental health support, such as lack of trust in others to maintain confidentiality, preference for dealing with issues independently, difficulties expressing their problems verbally, desire to keep negative feelings private, reluctance to burden or worry others (especially parents), and fear of getting into trouble with parents or teachers [27, 28]. In the study by Halliday and colleagues [33], participants mentioned that the lack of external support in specific circumstances led to feelings of isolation and disconnection from their usual support networks when friends and family faced additional pressures or when community volunteering opportunities to self-help diminished [33].

At the societal level, the perception of fear of stigmatisation, negative associations with being a “mental health patient” and concern about discrimination in the family and society was reported by several studies [7, 16, 22]. Some also feared being discriminated against, excluded, or judged negatively, with feelings of shame, embarrassment, a sense of being disgraced and engaging in self-blame [7, 15, 16, 24]. Similar perceptions were reported as barriers to seeking informal support with family and friends where participants were reluctant to disclose and discuss their mental health problems [16, 22–24]. Cultural barriers among certain ethnic groups of women, like losing personal and cultural identity, losing social acceptance, tolerating distress and isolation, and the practice of negotiating and accepting tensions to protect their *Izzat* (dignity), were reported as hindering factors for help-seeking behaviour for mental health issues [15].

At the institutional level, the long waiting time for the initial assessment, language barriers, insufficient addressing of mental health needs, presence of cultural naivety, insensitivity, and bias towards the needs of black and ethnic minorities service users and lack of awareness of different services among service users and providers, were also reported as barriers in seeking help from professional health services [7, 24, 25, 32]. Further, studies underlined the challenges and vulnerabilities that the ethnic minority population faces when expected to communicate openly about difficulties in the context of power dynamics that are already skewed towards white professionals [7, 16, 32]. Several articles illustrated that individuals from ethnic minority backgrounds were less likely to seek professional help for mental health [7, 15–17, 31, 33]. Some of these articles reported that individuals perceived a lack of equality, diversity, and inclusion within the mental health services systems to accommodate ethnic minority communities. In school, despite the acceptance of learning coping strategies from school-based initiatives, barriers that reduced adolescent engagement with HeadStart and CAMHS included a lack of friends’ participation, school staff strikes, and delayed initiation, leading to the discontinuation of the support and counselling activities [27].

### Findings from the Consultation Workshop

The participants in the workshop agreed with most of the findings presented in the scoping review. However, they highlighted some additional components that were not evidenced by the existing empirical research. The discussion highlighted the establishment of new mental health support teams (MHSTs) in school and college settings in partnership with the Department of Education. These teams include educational mental health practitioners who are trained to support schools in helping with providing informal mental health services. They believed that such changes within the educational settings had not been reported in the papers included in this review due to their recent implementation. However, it is important to understand whether these new school-based mental health promotion approaches are effective for those from minority ethnic backgrounds. Likewise, practitioners emphasised that despite the availability of online mental health helplines from the NHS and other organisations, it was not reflected adequately in the literature.

Participants shared and discussed the utilisation of different online mental health resources and digital platforms among these groups. Interestingly, Artificial Intelligence (AI) generated chat system “Question and Answer” was mentioned as one highly utilised resource as a coping strategy among young people. Likewise, the use of AI chatbots and improving access to psychological therapies (IAPT) to streamline mental health referrals was also discussed. Participants further highlighted that the evidence on IAPT among ethnic groups is important to understand.

In addition, participants cited the importance of the British Association for Counselling & Psychotherapy (BACP) as a helpful network for connecting the mental health system at different levels; however, this aspect has not been emphasised in the literature. Similarly, ‘Discovery College’, which is a growing assets-focused approach in supporting mental health and well-being, especially set up by people with lived experience to meet the needs of those living with mental health and learning disabilities, is equally crucial to explore among ethnic minorities. Interestingly, stakeholders expressed the belief that intergenerational differences in understanding mental health and well-being within the same family may also act as barriers to promoting mental health among ethnic minorities.

## Discussion

Our scoping review synthesises the identified evidence to highlight experiences and the use of coping strategies to enhance mental health and well-being among ethnic minority groups in the UK. By focusing on ethnic minorities, this review provides essential insight to inform considerations of ways to address the unmet mental health needs among ethnic minorities. Actions such as assisting individual life-skill development, strengthening community partnerships and improving access to support mechanisms, are likely to be important for addressing ethnicity-related mental health inequalities and helping people to overcome the adversity of mental health challenges.

This review highlights the self-help and immediate personal and social support network-based coping mechanisms practised among ethnic minorities, aligning with Folkman and Moskowitz’s [14] social aspects of coping. This describes the ways individuals utilise social and community support mechanisms to overcome their adverse circumstances, such as seeking support from parents, family members, siblings, friends, and partners, which were considered the first line of interaction in the process of coping mechanisms. It is widely acknowledged in various healthcare models that the family acts as an immediate resource for individual family members when they experience unfavourable health conditions [34]. Despite the increased ethnic diversity and cross-cultural partnerships in the UK in recent years, Britain’s minority groups still comprise a small proportion of the total population [35]. Because of such circumstances, most individuals from ethnic minority families often have strong familial connections and sociocultural ties with the members of their families and communities, which may induce them to choose their family members as a primary source of support while coping with their mental health issues [1]. The practice of family involvement in mental health issues in these groups shows positive alignment with the recommendations from the World Psychiatric Association [36] and the World Health Organisation’s Mental Health Action Plan (2013–2020) [37], which strongly emphasises the importance of family involvement and their collaborations in the delivery of the mental health services.

On the other hand, some adolescents cited challenges in seeking help with their immediate family and other networks, such as a lack of trust in others to keep secrets, a struggle to express their problems verbally, a desire to keep negative feelings private, a reluctance to burden or worry others (especially parents), and a fear of getting into trouble with parents or teachers [27, 28]. This is parallel to the findings from the review of studies conducted in the USA, where adolescents from immigrant families had higher chances of facing intergenerational stress and differences in practices of coping strategies [38]. As a result, young people were more likely to acculturate to Western culture quickly than their parents, so that they could put themselves in a wider, comfortable environment while dealing with stress and other forms of mental health issues [38, 39]. A study conducted among minority populations highlighted that cultural brokering needs to be practised with an adequate and nuanced understanding of cultural transformation so that families can capitalise on the positive aspect of their familial bond and cultural norms in the process of acculturation and cultural transformation [39]. Preservation of such cross-cultural communication skills can encourage young people to trust their family members and seek support from them while dealing with their mental health issues [39]. Moreover, the intergenerational transmission of culture plays a crucial role in creating a familial environment that promotes open discussion of mental health [40].

Likewise, religious or faith-based coping strategies, including practicing a spirituality-based approach, are aligned with Folkman and Moskowitz’s [14] religious coping strategies. Despite the expanding corpus of evidence that suggests greater religiosity is positively associated with better mental well-being [41–43], the situation may not always be true [44]. A household longitudinal study conducted in the UK found that those not affiliated with religiosity experienced better mental well-being than those strongly affixed to religiosity [44]. However, participation in religious activities may benefit in different ways, helping reduce loneliness, enhancing social support and improving networks, and encouraging individual engagement with other community services [21, 45, 46].

In addition, processing and expressing emotions through both physical and digital approaches was also found to be used as coping strategies among these groups, which aligned with Folkman and Moskowitz’s [14] emotional coping approach [14]. Different physical approaches to coping strategies were identified in our review. These findings are comparable with studies among other population groups and different countries’ contexts as well, where individuals were found to practice similar activities, including arts/crafts, music workshops, graffiti workshops, gardening, woodwork, meditation and reading for pleasure [47–52]. Processing and expressing emotions through creative actions have been widely researched, and the approach has shown potential as a mechanism to facilitate stress coping in various contexts and across all age groups of populations [47, 49, 50]. According to the theory of transformative coping, individuals practising those creative actions have a transformative quality that can help reduce negative emotions as well as transform negative emotions into a sustainable positive atmosphere and emotions [53, 54]. However, creative coping depends on the individual levels of skills and collective experiences to deal with different stressors in various circumstances [55]. Likewise, in some papers in the review, negative health behaviours such as alcohol consumption and smoking were also considered as creative coping strategies [26, 30, 32]. However, maladaptive behaviours were agreed to be unacceptable creative actions as they impair cognitive functions, negatively affecting overall well-being in the long term.

Despite the availability of community-based and professional mental health services such as GPs, psychiatrists, psychologists, guidance counsellors, and social workers within the formal mental healthcare systems, review results show that ethnic minorities face several challenges and barriers in accessing mental health services. Several articles illustrated that ethnic minority individuals were less likely to seek professional help for mental health [7, 15–17, 31, 33]. The findings can be explained by the fact that ethnic inequities in mental healthcare exist [2], where ethnic minorities have poor treatment outcomes of mental health services, poor competency in navigating through the healthcare system and, in some cases, long waiting times which further discourages ethnic minority population in seeking professional support from mental health services [2, 56, 57]. Also, the socio-economic disparities, cultural differences, and perceived inadequacy in cultural competency among health professionals were often linked to the poor utilisation of such services among ethnic minorities [2, 56, 57]. Conversely, the evidence shows that several community-based efforts, such as therapy-style sessions, peer-support groups, educational materials, gym access and a family services program, were implemented to facilitate access to services among ethnic minorities in the UK [57]. Studies show that translating educational materials into different languages has effectively overcome the structural barriers to accessing mental health services [58]. A study from Canada shows that health professionals receiving anti-stigma training have been found to have a positive influence on reducing preconceptions or stigma around certain cultural and ethnic groups and improving patient treatment experience [59].

Moving forward, signposting of services has been widely practiced in the UK through the social prescribing model [60]. However, there is limited evidence of how this approach best fits into practice, considering multiculturalism and the need for diverse ethnic groups [60]. Moreover, the evidence suggests that a social prescribing scheme embedded within community networks, with adequate consideration of cultural sensitivity, could significantly enhance patient trust and the usability of community mental health services [61]. However, results from multistakeholder consultation show that evidence of the social prescription model embedded within the wider ethnic and cross-cultural frameworks has been lacking among these groups. Additionally, evidence shows that strength-based approaches are proven to be effective in co-producing health services and promoting mental health in different contexts [62–65]. The strength-based approach focuses on the strengths and resources of the individual and community rather than on the people’s deficits [66]. This approach was also highlighted in our stakeholder discussion, where “Discovery College” was cited as one of the growing asset-focused approaches in supporting mental health. However, there is limited evidence that supports the application of an asset-focused approach among ethnic minorities. Effective application of such an approach could benefit by shifting the focus away from structural approaches to mental health interventions to adequately acknowledge the value, capacity, skills, knowledge, experiences, connections, and potential of ethnic minority individuals and communities [65, 66]. The application of this approach has also been widely reflected in evidence from the UK and other countries [58, 62–64, 66]. It is also clearly articulated in the UK’s health and social care guiding policy document [67]. However, the meaningful implication of such a multi-dimensional approach needs extensive research and resources to translate its essence into reality [65]. Further understanding of the coping strategies used among ethnic minority groups is needed to inform alternative approaches that will effectively address the mental health needs of ethnic minorities and reduce the mental health inequalities. To facilitate an interdisciplinary approach, greater insight is needed into the partnerships between families, schools and community networks involved in coping with mental ill-health.

### Strengths and Limitations

To the best of our knowledge, this review is the first to present synthesised evidence related to the practice of coping strategies to enhance mental health and well-being among ethnic minority groups in the UK. We conducted a stakeholder consultation workshop to validate the review findings among various stakeholders. This ensures that diverse experiences and expertise are brought to this review and increases the robustness of the results. The consultation has helped enrich and strengthen our research findings, providing directions for future research among ethnic minority groups. Thus, methodological aspects of our review emphasise the utilisation of stakeholder consultation as a mandatory step rather than an optional stage while conducting scoping reviews, as it enhances the relevance, clarity and transferability of the evidence. However, our review only includes empirical literature and does not include grey literature. The review is also limited to the specific population and the UK context; therefore, the findings may not apply to other settings. As with any review, our conclusions have been constrained by the availability and quality of the published evidence.

## Conclusions

Our review accumulated and synthesised the available empirical literature related to coping strategies and focused on identifying and presenting solutions to enhance mental health among ethnic minorities, rather than merely discussing barriers and challenges. The use of stakeholder consultation as the final step of the scoping review to validate the findings from the existing literature, and incorporating their reflections to highlight new initiatives, such as mental health support teams in schools and colleges, the use of digital platforms and artificial intelligence for mental health resources and the importance and need of asset-focused approach in supporting mental health and well-being of ethnic minority populations are some of the important findings. Despite the several efforts implemented for mental health promotion among ethnic minority groups, they are still struggling to access professional support services to manage their mental health issues effectively and lack adequate knowledge and skills on healthy coping strategies. This demonstrates the need for a deeper understanding of the ways that strategies and approaches used by ethnic minorities can be integrated to strengthen efforts to improve the mental health and well-being of minority ethnic populations in the UK and reduce ethnicity-related mental health inequalities.

## Data Availability

No datasets were generated or analysed during the current study.
